# Estimated trends in hospitalizations due to occupational injuries in Korea based on the Korean National Hospital Discharge In-depth Injury Survey (2006-2019)

**DOI:** 10.4178/epih.e2023042

**Published:** 2023-04-05

**Authors:** Seong-Uk Baek, Won-Tae Lee, Min-Seok Kim, Myeong-Hun Lim, Jin-Ha Yoon

**Affiliations:** 1 Department of Occupational and Environmental Medicine, Severance Hospital, Yonsei University College of Medicine, Seoul, Korea; 2 Institute for Occupational Health, Yonsei University College of Medicine, Seoul, Korea; 3 Graduate School, Yonsei University College of Medicine, Seoul, Korea; 4 Department of Public Health, Graduate School, Yonsei University, Seoul, Korea; 5 Department of Preventive Medicine, Yonsei University College of Medicine, Seoul, Korea

**Keywords:** Work-related injury, Industrial accident, Occupational disease, Workers, Workplace safety

## Abstract

**OBJECTIVES:**

In recent years, occupational injuries have sparked a huge social and political debate. Thus, in this study, we focused on the characteristics and trends of occupational injuries requiring hospitalization in Korea.

**METHODS:**

The Korea National Hospital Discharge In-depth Injury Survey was designed to estimate the annual number and characteristics of all injury-related hospitalizations in Korea. The annual number of hospitalizations due to occupational injuries and the age-standardized rates (ASRs) were estimated from 2006 to 2019. The annual percentage change (APC) and average annual percentage change (AAPC) of ASRs and their 95% confidence intervals (CIs) were calculated using joinpoint regression. All analyses were stratified by gender.

**RESULTS:**

In men, the APC of the ASRs of all-cause occupational injuries was -3.1% (95% CI, -4.5 to -1.7) in 2006-2015. However, a non-significant upward trend was observed after 2015 (APC, 3.3%; 95% CI, -1.6 to 8.5). In women, the APC of all-cause occupational injuries was -8.6% (95% CI, -12.1 to -5.1) in 2006-2012. However, a non-significant upward trend was observed after 2012 (APC, 2.1%; 95% CI, -0.9 to 5.2). A recent upward trend in stabbing injuries was observed after 2012 (APC, 4.7%; 95% CI, -1.8 to 11.8) in women. A non-significant overall increasing trend was also observed for occupational injuries caused by exposure to extreme temperatures (AAPC, 3.7%; 95% CI, -1.1 to 8.7) in women.

**CONCLUSIONS:**

A recent upward trend in all-cause injury hospitalizations and hospitalizations caused by stabbing injuries was observed. Therefore, active policy interventions are required to prevent occupational injuries.

## GRAPHICAL ABSTRACT

[Fig f3-epih-45-e2023042]

## INTRODUCTION

Working in a safe and healthy environment is a core right of all workers. The government and employers are obligated to ensure workers’ physical safety. However, many workers are still exposed to various physical hazards, including falls, entrapments, and exposure to extreme temperatures. Occupational injuries can cause a significant burden at the national level. Studies on disease burden over the past decades have suggested that injury-related risk factors are key elements in occupational exposure [1-3]. According to a previous article, in 2016, occupational injuries caused approximately 332,500 deaths globally (21.7% of total occupational-attributable deaths) and 21.4 million disability-adjusted life years (DALYs) [1]. In the United States, the annual economic cost of occupational injuries was estimated to be US dollar (USD) 192 billion [4]. Occupational injuries often diminish quality of life and can cause mental aftereffects even after treatment [5,6]. Furthermore, occupational injuries lead to a loss of labor, thereby causing a subsequent decrease in income [7].

In recent years, occupational injuries have sparked significant social and political debates. In particular, workers with precarious employment, such as subcontractors or temporary employees, are more likely to experience occupational injuries, thus raising concerns about social inequality [8,9]. Governments and companies are now being asked to implement additional active policies to prevent occupational injuries. For example, the Korean National Assembly introduced a series of legislation aimed at preventing occupational injuries [10]. A multi-dimensional approach was also proposed to reduce occupational injuries, such as the creation of a safety culture in organizations [11,12].

To prevent occupational injuries, their current status and trends should first be examined. Globally, the number of deaths and DALYs due to occupational injuries has decreased by more than 40% over the last 20 years, and this decrease could be attributed to improvements in working environments and developments in industrial safety technology [1]. Similarly, efforts have been made to understand the status and trends of occupational injuries in Korea [13,14]. For instance, Kang & Kwon [13] reported that occupational injuries in Korean wage workers gradually decreased in the 2000s. However, increasing trends in occupational injuries have been identified in certain industries, including construction and agriculture [14].

Nevertheless, the current literature has the following limitations. First, no study has been published on recent trends in occupational injuries (i.e., after 2010) in Korea. Second, previous studies have investigated occupational injuries on the basis of data from the Industrial Accident Compensation Insurance (IACI). However, given that the IACI did not cover self-employed individuals or freelancers until recently, the cases reported in the IACI did not represent the number of occupational injuries among all workers. For example, the IACI included approximately 12 million workers in 2010 [14], which accounted for approximately 58.3% of the total 24 million workers in Korea [15].

The Korea National Hospital Discharge In-depth Injury Survey (KNHDIS) was designed to estimate the number and characteristics of all injury-related hospitalizations in Korea [16]. Recent studies have explored trends in all-cause injury-related hospitalizations of the entire population in Korea using the KNHDIS [17,18]. In the current study, we focused on the characteristics and trends of hospitalizations due to occupational injuries in Korea. We believe that our findings will contribute to the literature by providing useful information that can help policymakers implement proper interventions to prevent occupational injuries in Korea.

## MATERIALS AND METHODS

### Data source

Our study analyzed raw data from the KNHDIS, which has been conducted annually by the Korea Disease Control and Prevention Agency (KDCA) since 2004 [16]. The KDCA launched the KNHDIS to generate statistical data on the epidemiological characteristics of injury-related hospitalizations in Korea from hospitals’ medical records. The target population of the KNHDIS includes all patients who have been discharged from general hospitals in Korea with 100 beds or more, excluding specialized hospitals. The KNHDIS employs multi-staged stratified cluster sampling. Sample hospitals are selected according to the region and number of beds, and approximately 9% of all discharged patients from the sampled hospitals are selected as sample patients. Therefore, the KNHDIS contains a multi-year, cross-sectional dataset that allows multiple admissions of the same patient. The survey items include information on patient demographic features (gender, age, and region) and are coded for principal or additional diagnoses on the basis of the Korean Standard Classification of Disease version 7 (KCD-7), which corresponds to the International Classification of Diseases (10th revision), and the mechanisms and types of injuries.

The number of participating hospitals gradually increased from 147 in 2004 to 206 in 2019, and the number of sampled patients increased from 175,948 in 2004 to 318,547 in 2019. Among the sampled patients, the number of injury-related cases accounted for approximately 13-15% of the total discharged cases. Given that the classification of injury mechanisms has remained constant since 2006, cases from 2006 to 2019 were included in this study. We limited our sample to injury-related cases in patients aged between 15 years and 64 years (266,547 cases), who are considered the economically active population in Korea

### Injury-related hospitalizations

Injury-related cases were defined as those with codes S00-T98 (injury, poisoning, and certain other consequences of external causes) as their primary or additional diagnoses according to the KCD-7. However, cases with codes T78, T80-T88, T90, or Y40-Y98 were excluded because information on the mechanisms of these cases was not investigated and because the meaning of the injuries was unclear. For all injury-related cases, codes for external causes of injury, namely, V01-Y98 (external causes of morbidity and mortality), were collected.

In all injury-related cases, except for cases with codes T78, T80-T88, T90, or Y40-Y98, information about the external causes of injuries (e.g., intent, place of occurrence, activity at the time of injury, and mechanism of the injury) was collected on the basis of in-depth medical record investigations by trained professionals. Based on an in-depth review of medical records, the KNHDIS classifies activities when injured as exercise, leisure activities, work with income, work without income (e.g., volunteering or housework), education, travel, daily life, treatment, other activities, and unknowns. An occupational injury was defined as the occurrence of injuries while engaging in work with income.

Finally, the mechanisms of injuries were classified into traffic accidents, falls, collisions (struck by/against), poisoning, stabbing, exposure to extreme temperatures, poisoning, and others (sexual assault, gunshot, asphyxiation, drowning, others, and unknown) according to the KCD-7 codes. Supplementary Material 1 presents the classification of the injury mechanisms according to the KCD-7.

### Statistical analysis

The KNHDIS is a complex-sample survey, and sample weights were applied to each case. Our study employed complex-sample frequency analysis to estimate the weighted number of hospital admission due to injuries and reflected the 2-staged stratified clustered sampling used in the KNHDIS [16]. The SURVEYMEANS procedure (SAS Institute Inc., Cary, NC, USA) was used to estimate the number of hospitalizations. Thereafter, the crude rate of hospitalization per 100,000 total population or workers was calculated by dividing the estimated number of hospitalizations by the total population or working population in Korea each year [15]. Direct standardization was then employed to calculate the agestandardized rate (ASR) using the age distribution of the working population of 2019 as the reference population. Age standardization was conducted using 5-year units of age from 15 years to 64 years. Finally, we analyzed trends in the ASR by using joinpoint regression with a log-linear model [19]. Joinpoint regression makes it possible to detect a change in the trend of the variable of interest during the study period. The most parsimonious models for each gender and injury mechanism were automatically selected to best fit the data via permutation tests [19]. The annual percentage change (APC) of the ASR and its 95% confidence intervals (CIs) were calculated to analyze the yearly trends of each injury type. In addition, the average annual percentage change (AAPC) was calculated to measure the trend of hospitalization due to injuries during the overall study period (2006-2019). Joinpoint version 4.9.1.0 (National Cancer Institute, Bethesda, MD, USA) was used to perform the trend analyses. All analyses were gender-stratified. Visualization was performed using R version 4.2.1 (R Foundation for Statistical Computing, Vienna, Austria). Statistical significance was defined as p-value< 0.05.

### Ethics statement

This study was reviewed and approved by the Institutional Review Board (IRB) of Yonsei Health System (No. 4-2022-1396). The requirement for informed consent was waived by the IRB.

## RESULTS

Table 1 presents the estimated number of hospitalizations due to injuries and the crude rate per 100,000 Korean population. In 2019, the estimated numbers of hospitalizations due to occupational injuries were 75,560 and 15,314 for men and women, respectively; these values accounted for approximately 18.9% and 6.0% of all-cause injury-related hospitalizations, respectively.

Table 2 presents the estimated number of occupational injury-related hospitalization cases and crude rates per 100,000 workers according to age group and injury mechanisms in 2006 and 2019. In men, the total estimated number decreased from 85,621 to 75,560, and the crude rate of hospitalization due to occupational injuries decreased from 673 to 537 (per 100,000 workers). In women, the total estimated number decreased from 17,765 to 15,314, and the crude rate of hospitalization due to occupational injuries decreased from 195 to 145 (per 100,000 workers). Although the crude rate of hospitalization due to occupational injuries decreased in most age groups, increases in the hospitalization rates were observed for both men and women among workers aged 60-64 years. Falls and collisions (struck by/against) were the most common injury mechanisms in occupational injuries. However, the estimated crude rate of fall injuries among men decreased from 161 to 157 (per 100,000 workers). In addition, the estimated crude rate of fall injuries among women decreased from 59 to 54 (per 100,000 workers). Similarly, the estimated crude rate of collision (struck by/against) injuries among men decreased from 261 to 206 (per 100,000 workers). In addition, the estimated crude rate of collision (struck by/against) injuries among women decreased from 60 to 32 (per 100,000 workers). By contrast, the estimated crude rate of hospitalization due to occupational exposure to extreme temperatures increased from 14 to 26 (per 100,000 workers) among men and increased from 7 to 23 (per 100,000 workers) among women. Supplementary Materials 2 and 3 show the estimated number of hospitalizations due to occupational injuries and their crude rates for all study years from 2006 to 2019.

Figure 1 and Table 3 present the ASRs and trends in occupational injuries according to the mechanisms among men. The APC of all-cause occupational injuries was -3.1% (95% CI, -4.5 to -1.7) from 2006 to 2015. However, a non-significant upward trend was observed after 2015 (APC, 3.3%; 95% CI, -1.6 to 8.5). Statistically significant downward trends were observed for occupational injuries caused by traffic accidents (AAPC, -6.3%; 95% CI, -8.5 to -4.1), collision (struck by/against) (AAPC, -1.8%; 95% CI, -2.8 to -0.8), and poisoning (AAPC, -3.5%; 95% CI, -4.9 to -2.1).

Figure 2 and Table 3 present the ASRs and trends in occupational injuries according to the mechanisms among women. The APC of all-cause occupational injury was -8.6% (95% CI, -12.1 to -5.1) in 2006-2012. However, a non-significant upward trend was observed after 2012 (APC, 2.1%; 95% CI, -0.9 to 5.2). Statistically significant downward trends were observed for occupational injuries caused by traffic accidents (AAPC, -10.6%; 95% CI, -14.5 to -6.5), collision (struck by/against) (APC, -4.4%; 95% CI, -6.3 to -2.5), and stabbing (APC, -5.1%; 95% CI, -8.9 to -1.0).

## DISCUSSION

Occupational injuries are of great concern worldwide. Therefore, preventing occupational injuries is a major challenge for both epidemiologists and policymakers. To the best of our knowledge, this study is the first to explore occupational injuries among all workers in Korea, including those not covered by the IACI. This study investigated the estimated number and yearly trends of occupational injuries on the basis of the KNHDIS data. Our findings suggest that the estimated number of total hospitalizations due to injuries and the hospitalization rates decreased during the overall period; however, different trends were observed for each injury mechanism.

A study on the disease burden in Korea estimated the annual economic cost of all-cause injuries to be USD25.4 billion annually [20]. Our estimations suggest that occupational injuries account for approximately 19% and 6% of total injury-related hospitalizations in Korea for men and women, respectively. In addition, the total estimated number of hospitalizations due to all-cause occupational injuries tended to decrease before 2012-2015 but stagnated or increased after then. Considering that occupational injuries have received significant political and social attention over the past decades, various policies have been implemented to prevent them [21]. However, the current study emphasizes that occupational injuries still cause a high socioeconomic burden in Korea. Nevertheless, an overall decreasing trend in occupational injuries was observed from 2006 to 2019. This is contrary to previous studies showing that the all-cause hospitalization rate in the entire population steadily increased from 2004 to 2016 in Korea [16,18]. The opposite trends in the hospitalization rates of occupational injuries and the total number of injuries suggest that policy interventions for the prevention of work-related accidents may have been effective for specific types of injuries (e.g., traffic accidents).

Our analysis estimates that 90,874 hospitalizations due to occupational injuries occurred among 24.5 million total workers in 2019 (370 cases per 100,000 workers). According to the annual report by the Ministry of Employment and Labor of Korea, a total of 109,242 occupational accidents or diseases occurred in 18.7 million workers covered by IACI in 2019; among these accidents, 94,047 were classified as occupational accidents (502 cases per 100,000 workers) [22]. The overall underestimation in our analysis may be attributable to the fact that all outpatients were excluded from our sample. Nevertheless, we were able to identify annual trends in occupational injuries requiring hospitalization.

The distribution of occupational injuries observed in this present study is in line with the findings from the annual IACI report. For instance, according to the IACI report, falls or collisions (struck by/against) were the 2 most common occupational injury mechanisms [22]. Our current study is also in line with the IACI report regarding the finding that the industrial accident rate has become stagnant or increased since 2015 [22]. It is also noteworthy that the number of fatal injuries due to traffic accidents that occurred inside or outside the workplace has steadily decreased, according to the industrial accident statistics in Korea [23].

Previous studies have shown that the incidence of occupational injuries steadily decreased until 2010 in Korea [13,14]. The current study observed an overall decreasing trend in total occupational injuries during the study period (2006-2019). However, a recent upward trend was observed in all-cause injury-related hospitalizations. In an analysis according to the specific injury mechanisms, we found that the recent increase in the ASRs of falls and stabbing injuries contributed to this trend. An earlier study that analyzed trends in injury hospitalization rates in the entire population also reported an increase in hospitalization rates due to falls [17]. In addition, occupational stabbing injuries have increased since 2012, particularly among women. There has also been a non-significant increase in the ASR of hospitalization due to occupational exposure to extreme temperatures. Therefore, our findings imply the need for active interventions to prevent injuries caused by falls, slips, cuts, stabbings, and burns.

A steady decrease in the hospitalization rate of injuries caused by traffic accidents and collisions (struck by/against) was observed in both men and women. A study reported that approximately 15% of traffic accidents occurred during work in Korea [24]. In this study, traffic accidents that occurred during commuting to and from workplaces were also categorized as occupational injuries. Park et al. [17] observed that hospitalizations due to traffic accidents steadily decreased in the entire population of Korea. In addition, injury-related mortality from traffic accidents in Korea has been steadily decreasing since the mid-1990s, suggesting that road safety policies have been effective in preventing injury-related hospitalizations in the entire population [25].

Caution is needed when interpreting the results of this study because of the following limitations. First, the estimated number of hospitalizations in our study did not include all occupational injuries that occurred in Korea. Most importantly, outpatients were excluded from this study. People hospitalized in certain hospitals specializing in the hand, digits, joint, or spine and hospitals with fewer than 100 beds were excluded. Therefore, our findings should not be interpreted as accurately estimating the total number of occupational injuries in Korea. However, the purpose of this study was to estimate the overall trend in occupational injuries during the study period. Second, the KNHDIS does not collect detailed occupational characteristics of injured workers, such as occupation, industrial sector, and employment type (employee, self-employed, or others); therefore, further in-depth research is needed to investigate the risk of occupational injuries according to occupation or industry. Third, in this study, occupational injuries were classified using a retrospective review of medical records. Therefore, misclassifications may occur if the medical staff does not describe the injury that occurred during work in the medical record. Fourth, the KNHDIS has the following limitations in monitoring occupational injuries: Compared to the IACI, which reports all cases approved for medical care due to an occupational injury, outpatient injured patients are excluded, and total injured cases are predicted based on the sampling method. In addition, there is a possibility of misclassification of occupational injuries. We expect further research to overcome these issues by introducing methods to estimate outpatient cases and more precisely classify occupational injuries.

Despite these limitations, our study is the first to estimate the recent trend in hospitalizations due to occupational injuries among all workers. In addition, given that the KNHDIS contains information on the external causes of injuries, it was possible to estimate the trend according to each injury mechanism.

In conclusion, the estimated age-adjusted hospitalization rates due to all-cause occupational injuries showed a steadily decreasing trend until 2012 for men and 2015 for women; however, a non-significant increasing trend was later observed. Hospitalizations due to occupational injuries caused by traffic accidents and collisions (struck by/against) continued to decrease during the observation period (2006-2019). However, a recent upward trend in hospitalizations due to occupational injury caused by falls and stabbings was observed. Therefore, active policy interventions are required to prevent occupational injuries.

## Figures and Tables

**Figure 1.**
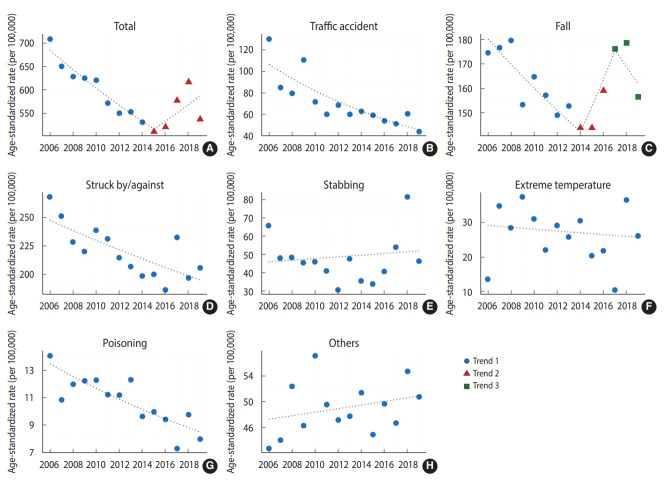
Age-adjusted rate of occupational injury hospitalizations by injury mechanism for men. (A) total, (B) traffic accidents, (C) falls, (D) struck by/against, (E) stabbing, (F) extreme temperature, (G) poisoning, and (H) others.

**Figure 1.**
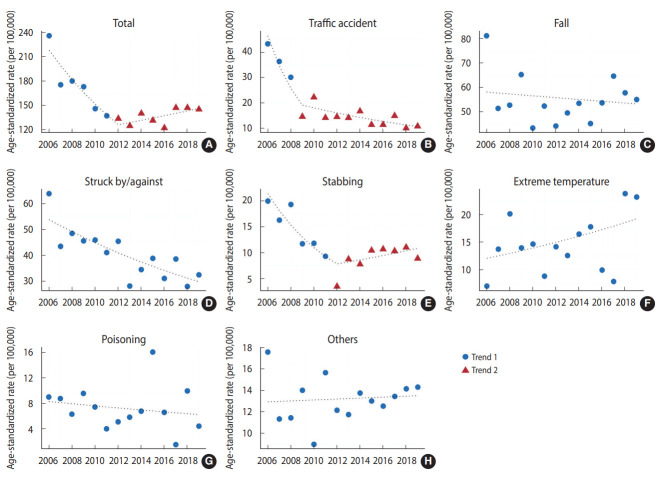
Age-adjusted rate of occupational injury hospitalizations by injury mechanism for women. (A) total, (B) traffic accidents, (C) falls, (D) struck by/against, (E) stabbing, (F) extreme temperature, (G) poisoning, and (H) others.

**Figure f3-epih-45-e2023042:**
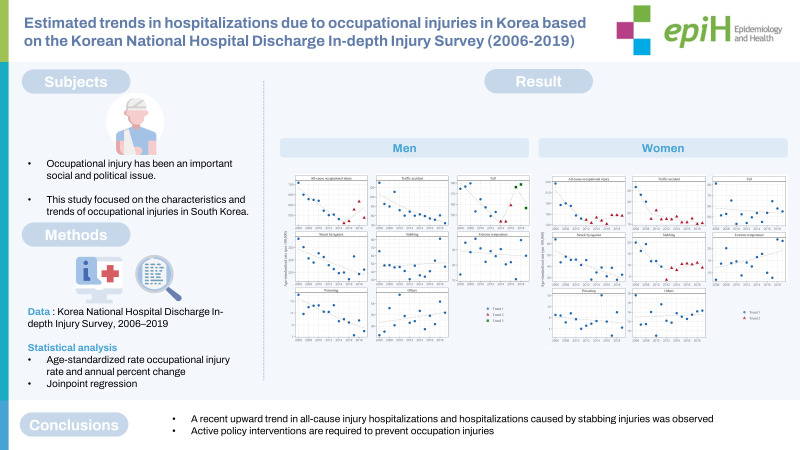


**Table 1.** The observed and estimated hospitalization cases of all-cause and occupational injuries

Year	Men				Women			
	Estimated cases		Crude rates^1^		Estimated cases		Crude rates^1^	
	All-cause injury	Occupational injury	All-cause injury	Occupational injury	All-cause injury	Occupational injury	All-cause injury	Occupational injury
2006	414,997	85,621	1,703	351	213,587	17,765	887	74
2007	423,426	80,183	1,729	327	225,418	13,492	932	56
2008	422,755	77,508	1,714	314	233,363	14,254	957	58
2009	436,772	77,036	1,763	311	242,549	13,506	989	55
2010	470,217	79,049	1,890	318	262,807	12,369	1,065	50
2011	464,360	73,766	1,852	294	263,036	11,774	1,058	47
2012	481,842	72,091	1,913	286	268,227	11,309	1,072	45
2013	475,238	73,134	1,880	289	275,195	11,200	1,094	45
2014	463,471	72,094	1,821	283	270,981	13,031	1,071	52
2015	469,260	70,034	1,834	274	282,900	12,689	1,113	50
2016	443,452	72,232	1,727	281	277,097	11,966	1,085	47
2017	449,306	81,058	1,746	315	282,049	14,853	1,101	58
2018	418,961	86,386	1,620	334	275,404	15,159	1,070	59
2019	399,661	75,560	1,540	291	256,959	15,314	995	59

1 The estimated number of injuries per 100,000 Korean population.

**Table 2.** The estimated number and crude rate of occupational injury-related hospitalizations according to age groups and injury mechanisms in 2006 and 2019

Characteristics		Men				Women			
		2006		2019		2006		2019	
		Cases	Crude rate^1^	Cases	Crude rate^1^	Cases	Crude rate^1^	Cases	Crude rate^1^
Total		85,621	673	75,560	537	17,765	195	15,314	145
Age (yr)									
	15-19	1,244	1,414	697	766	273	235	199	186
	20-24	5,339	980	3,653	696	702	76	629	84
	25-29	8,202	573	4,816	369	1,021	83	542	46
	30-34	9,210	481	5,167	348	811	77	475	47
	35-39	12,949	636	6,008	320	2,119	170	714	62
	40-44	12,033	616	6,838	383	1,837	140	890	75
	45-49	12,331	658	11,003	536	3,621	279	1,457	98
	50-54	10,617	786	10,615	565	3,048	342	3,311	229
	55-59	8,025	882	14,440	804	2,867	488	3,540	268
	60-64	5,670	915	12,323	976	1,465	338	3,558	395
Mechanism									
	Traffic accident	15,421	121	6,190	44	3,135	35	1,059	10
	Falls	20,423	161	22,010	157	5,338	59	5,735	54
	Struck by/against	33,146	261	28,914	206	5,476	60	3,398	32
	Stabbing	7,913	62	6,515	46	1,545	17	830	8
	Extreme temperature	1,786	14	3,675	26	647	7	2,434	23
	Poisoning	1,509	12	1,120	8	584	6	365	3
	Others	5,424	43	7,136	51	1,039	11	1,493	14

1 The estimated number of occupational injuries per 100,000 workers.

**Table 3.** Trends in age-adjusted hospitalization rates due to occupational injuries among men and women

Mechanism		Trend 1		Trend 2		Trend 3		Overall trend	
		Period	AAPC, % (95% CI)	Period	AAPC, % (95% CI)	Period	AAPC, % (95% CI)	Period	AAPC, % (95% CI)
Men									
	All-cause	2006-2015	-3.1 (-4.5, -1.7)	2015-2019	3.3 (-1.6, 8.5)	-	-	2006-2019	-1.2 (-2.7, 0.4)
	Traffic accidents	-	-	-	-	-	-	2006-2019	-6.3 (-8.5, -4.1)
	Falls	2006-2014	-2.9 (-4.5, -1.3)	2014-2017	7.3 (-6.9, 23.6)	2017-2019	-4.0 (-15.9, 9.5)	2006-2019	-0.8 (-3.9, 2.4)
	Collisions (struck by/against)	-	-	-	-	-	-	2006-2019	-1.8 (-2.8, -0.8)
	Stabbing	-	-	-	-	-	-	2006-2019	
	Extremetemperature	-	-	-	-	-	-	2006-2019	
	Poisoning	-	-	-	-	-	-	2006-2019	
	Others	-	-	-	-	-	-	2006-2019	
Women									
	All-cause	2006-2012	-8.6 (-12.1, -5.1)	2012-2019	2.1 (-0.9, 5.2)			2006-2019	-3.0 (-5.0, -1.0)
	Traffic accidents	2006-2009	-25.4 (-37.8, -10.5)	2009-2019	-5.6 (-9.2, -1.8)			2006-2019	-10.6 (-14.5, -6.5)
	Falls	-	-	-	-			2006-2019	-0.7 (-3.0, 1.8)
	Collisions (struck by/against)	-	-	-	-			2006-2019	-4.4 (-6.3, -2.5)
	Stabbing	2006-2012	-15.3 (-21.2, -9.0)	2012-2019	4.7 (-1.8, 11.8)			2006-2019	-5.1 (-8.9, -1.0)
	Extremetemperature	-	-	-	-			2006-2019	3.7 (-1.1, 8.7)
	Poisoning	-	-	-	-			2006-2019	-2.2 (-6.7, 2.6)
	Others	-	-	-	-			2006-2019	0.3 (-1.8, 2.5)

APC, annual percentage change; CI, confidence interval; AAPC, average annual percentage change.

## SUPPLEMENTARY MATERIALS

Supplementary Material 1. Classification of injury mechanisms according to the Korean Standard Classification of Disease Version 7 (KCD-7)

Supplementary Material 2. Estimated number of hospitalization due to occupational injury according to age group and injury mechanism among men workers

Supplementary Material 3. Estimated number of hospitalization due to occupational injury according to age group and injury mechanism among women workers
