# Supplementary material for: Estimated trends in hospitalizations due to occupational injuries in Korea based on the Korean National Hospital Discharge In-depth Injury Survey (2006-2019)
Source: Epidemiol Health. 2023 Apr 5;45:e2023042. doi: 10.4178/epih.e2023042 (PMC10396798; doi:10.4178/epih.e2023042)
Supplement: Supplementary Material 1. — Classification of injury mechanisms according to the Korean Standard Classification of Disease Version 7 (KCD-7) [file epih-45-e2023042-Supplementary-1.docx]

**Supplementary Material 1.** Classification of injury mechanisms according to the Korean Standard Classification of Disease Version 7 (KCD-7)

|  | Korean Standard Classification of Disease | | | | | |
| --- | --- | --- | --- | --- | --- | --- |
| Mechanism | Accidents  (V01-X59) | Intentional self-harm  (X60-X84) | Assault  (X85-Y09) | Event of undetermined intent  (Y10-Y34) | Legal intervention (Y35) | Operations of war (Y36) |
| Traffic accident | V01-V99 | X82 | Y03 | Y32 |  | Y35.1 |
| Falls | W00-W19 | X80 | Y01 | Y30 |  |  |
| Struck by/against | W20-W24, W27-W31, W35-W40, W45, W49-W52, W54-W64 | X79 | Y00, Y04 | Y29 | Y35.3 |  |
| Stabbing | W25-W27, W29, W45, W49, W53, W54-W64 | X78 | X99 | Y28 | Y35.(4, 5) |  |
| Extreme temperature | X00-X19, X32 | X76-X77 | X97-X98 | Y26-Y27 |  | Y36.3 |
| Poisoning | X20-X29, X40-X49 | X60-X69 | X85-X90 | Y10-Y19 | Y35(2, 5) |  |
| Others | W32-W34, W35-W44, W46, W49 W75-W84, W65-W74 | X70, X71, X72-X74, X75, X76-X77, X81 | X91, X92, X93-X95, X96, Y02 | Y20, Y21, Y22-Y24, Y25, Y31 | Y35.(1, 2), Y35.(0, 5), Y35(2, 5) | Y36.0, Y36.4 |
