# Supplementary material for: Estimated trends in hospitalizations due to occupational injuries in Korea based on the Korean National Hospital Discharge In-depth Injury Survey (2006-2019)
Source: Epidemiol Health. 2023 Apr 5;45:e2023042. doi: 10.4178/epih.e2023042 (PMC10396798; doi:10.4178/epih.e2023042)
Supplement: Supplementary Material 2. — Estimated number of hospitalization due to occupational injury according to age group and injury mechanism among men workers [file epih-45-e2023042-Supplementary-2.docx]

**Supplementary Material 2.** Estimated number of hospitalization due to occupational injury according to age group and injury mechanism among men workers

|  | **2006** | **2007** | **2008** | **2009** | **2010** | **2011** | **2012** | **2013** | **2014** | **2015** | **2016** | **2017** | **2018** | **2019** |
| --- | --- | --- | --- | --- | --- | --- | --- | --- | --- | --- | --- | --- | --- | --- |
| **Age group** |  |  |  |  |  |  |  |  |  |  |  |  |  |  |
| 15-19 | 1244 (202) | 928 (140) | 1163 (203) | 1451 (288) | 800 (138) | 817 (122) | 1035 (149) | 1333 (192) | 905 (116) | 1125 (146) | 1029 (130) | 650  (81) | 944 (148) | 697 (109) |
| 20-24 | 5339 (140) | 4739 (134) | 3652 (118) | 3378 (112) | 3396 (114) | 2896 (93) | 3091 (90) | 2497 (71) | 3497 (92) | 3652 (91) | 2804 (71) | 3741 (97) | 4355 (123) | 3653 (99) |
| 25-29 | 8202 (82) | 8375 (83) | 6127 (61) | 7267 (74) | 6966 (73) | 5813 (63) | 4883 (56) | 4356 (53) | 4992 (61) | 3596 (44) | 4040 (48) | 4929 (59) | 5713 (64) | 4816 (53) |
| 20-34 | 9210 (69) | 8698 (67) | 7697 (61) | 6647 (55) | 7379 (61) | 7027 (57) | 6412 (50) | 6552 (51) | 6912 (54) | 5370 (43) | 5936 (51) | 6633 (60) | 6160 (58) | 5167 (50) |
| 35-39 | 12949 (91) | 10666 (74) | 9884 (68) | 9127 (64) | 8900 (63) | 8223 (60) | 6721 (50) | 6988 (54) | 5763 (45) | 5546 (44) | 6693 (52) | 7126 (53) | 7301 (55) | 6008 (46) |
| 40-44 | 12033 (88) | 11193 (82) | 10899 (80) | 11242 (82) | 11718 (84) | 9379 (65) | 9973 (68) | 9216 (63) | 7945 (54) | 7629 (53) | 8780 (63) | 8083 (60) | 7161 (56) | 6838 (55) |
| 45-49 | 12331 (94) | 12443 (92) | 13962 (101) | 12098 (88) | 13573 (99) | 10951 (81) | 11048 (82) | 11740 (87) | 12159 (88) | 11472 (82) | 9919 (69) | 10221 (70) | 11568 (79) | 11003 (77) |
| 50-54 | 10617 (112) | 10039 (100) | 10846 (100) | 11842 (103) | 12613 (104) | 13215 (104) | 12384 (93) | 12264 (90) | 11500 (85) | 11521 (85) | 11010 (84) | 13639 (104) | 13347 (104) | 10615 (81) |
| 55-59 | 8025 (126) | 7812 (114) | 8515 (122) | 8840 (121) | 8461 (107) | 9761 (113) | 10982 (118) | 11150 (111) | 11628 (106) | 12536 (110) | 13008 (108) | 14932 (119) | 17321 (136) | 14440 (115) |
| 60-64 | 5670 (131) | 5289 (118) | 4764 (103) | 5143 (106) | 5243 (103) | 5685 (104) | 5561 (98) | 7040 (119) | 6792 (108) | 7586 (112) | 9013 (123) | 11105 (139) | 12516 (149) | 12323 (139) |
| **Mechanism** |  |  |  |  |  |  |  |  |  |  |  |  |  |  |
| All-cause | 85621 (673) | 80183 (622) | 77508 (597) | 77036 (594) | 79049 (600) | 73766 (549) | 72091 (530) | 73134 (534) | 72094 (517) | 70034 (499) | 72232 (513) | 81058 (572) | 86386 (614) | 75560 (537) |
| Traffic accident | 15421 (121) | 10261 (80) | 9597 (74) | 13329 (103) | 8843 (67) | 7587 (56) | 8794 (65) | 7810 (57) | 8466 (61) | 7958 (57) | 7551 (54) | 7158 (51) | 8510 (60) | 6190 (44) |
| Falls | 20423 (161) | 20753 (161) | 20867 (161) | 18226 (141) | 20454 (155) | 19669 (146) | 19029 (140) | 19941 (146) | 19126 (137) | 19373 (138) | 21760 (155) | 24619 (174) | 25045 (178) | 22010 (157) |
| Struck by/against | 33146 (261) | 32024 (248) | 28547 (220) | 27561 (212) | 30603 (232) | 30220 (225) | 28645 (211) | 27293 (199) | 27414 (196) | 27710 (197) | 25826 (184) | 32694 (231) | 27511 (195) | 28914 (206) |
| Stabbing | 7913 (62) | 5989 (46) | 6439 (50) | 5750 (44) | 5968 (45) | 5560 (41) | 4053 (30) | 6281 (46) | 4704 (34) | 4628 (33) | 5727 (41) | 7581 (54) | 11402 (81) | 6515 (46) |
| Extreme temperature | 1786 (14) | 4409 (34) | 3818 (29) | 4706 (36) | 4128 (31) | 2865 (21) | 3884 (29) | 3414 (25) | 4251 (30) | 2915 (21) | 3119 (22) | 1523 (11) | 5081 (36) | 3675 (26) |
| Poisoning | 1509 (12) | 1064  (8) | 1435 (11) | 1519 (12) | 1558 (12) | 1299 (10) | 1419 (10) | 1681 (12) | 1078  (8) | 1178  (8) | 1254  (9) | 890  (6) | 1178  (8) | 1120  (8) |
| Others | 5424 (43) | 5683 (44) | 6805 (52) | 5944 (46) | 7495 (57) | 6567 (49) | 6267 (46) | 6715 (49) | 7055 (51) | 6272 (45) | 6995 (50) | 6591 (47) | 7660 (54) | 7136 (51) |

* Values are presented as estimated number (hospitalization rate per 100,000 worker). * Values are presented as estimated number (hospitalization rate per 100,000 worker).
