# Supplementary material for: Estimated trends in hospitalizations due to occupational injuries in Korea based on the Korean National Hospital Discharge In-depth Injury Survey (2006-2019)
Source: Epidemiol Health. 2023 Apr 5;45:e2023042. doi: 10.4178/epih.e2023042 (PMC10396798; doi:10.4178/epih.e2023042)
Supplement: Supplementary Material 3. — Estimated number of hospitalization due to occupational injury according to age group and injury mechanism among women workers [file epih-45-e2023042-Supplementary-3.docx]

**Supplementary Material 3.** Estimated number of hospitalization due to occupational injury according to age group and injury mechanism among women workers

|  | **2006** | **2007** | **2008** | **2009** | **2010** | **2011** | **2012** | **2013** | **2014** | **2015** | **2016** | **2017** | **2018** | **2019** |
| --- | --- | --- | --- | --- | --- | --- | --- | --- | --- | --- | --- | --- | --- | --- |
| **Age group** |  |  |  |  |  |  |  |  |  |  |  |  |  |  |
| 15-19 | 273 (34) | 36 (5) | 88 (12) | 161 (21) | 123 (14) | 117 (13) | 230 (25) | 143 (16) | 232 (25) | 135 (14) | 73 (8) | 284 (30) | 210 (26) | 199 (27) |
| 20-24 | 702 (11) | 459 (8) | 674 (12) | 547 (11) | 560 (11) | 604 (12) | 334 (6) | 475 (9) | 809 (15) | 617 (11) | 340 (6) | 373 (7) | 878 (16) | 629 (12) |
| 25-29 | 1021 (12) | 1128 (13) | 718 (8) | 819 (9) | 798 (9) | 554 (7) | 507 (6) | 530 (7) | 452 (6) | 630 (9) | 306 (4) | 683 (9) | 600 (8) | 542 (7) |
| 20-34 | 811 (11) | 916 (13) | 736 (11) | 569 (9) | 548 (8) | 390 (5) | 332 (4) | 407 (5) | 366 (5) | 608 (8) | 455 (6) | 438 (6) | 443 (6) | 475 (7) |
| 35-39 | 2119 (24) | 863 (10) | 1519 (17) | 1032 (12) | 1274 (16) | 873 (11) | 520 (7) | 851 (12) | 510 (7) | 394 (6) | 721 (9) | 485 (6) | 741 (9) | 714 (9) |
| 40-44 | 1837 (20) | 1505 (16) | 1654 (18) | 1372 (15) | 1278 (13) | 1389 (14) | 719 (7) | 1081 (11) | 1262 (13) | 662 (7) | 1057 (12) | 1308 (15) | 1013 (12) | 890 (11) |
| 45-49 | 3621 (40) | 2750 (29) | 2458 (25) | 2471 (26) | 2319 (25) | 1709 (18) | 2243 (24) | 1489 (16) | 1704 (17) | 1574 (15) | 875 (8) | 1569 (14) | 2156 (20) | 1457 (14) |
| 50-54 | 3048 (49) | 2329 (35) | 2710 (37) | 2560 (33) | 2636 (32) | 2698 (30) | 2338 (26) | 2628 (27) | 2937 (30) | 3122 (32) | 3013 (32) | 3034 (32) | 3052 (32) | 3311 (33) |
| 55-59 | 2867 (70) | 2041 (47) | 2056 (45) | 1993 (42) | 1803 (35) | 2431 (42) | 2055 (33) | 1757 (26) | 2518 (34) | 3168 (40) | 2541 (30) | 3468 (39) | 3444 (38) | 3540 (38) |
| 60-64 | 1465 (48) | 1464 (48) | 1640 (54) | 1982 (63) | 1030 (32) | 1009 (30) | 2032 (55) | 1838 (47) | 2242 (54) | 1778 (38) | 2586 (50) | 3211 (59) | 2620 (46) | 3558 (56) |
| **Mechanism** |  |  |  |  |  |  |  |  |  |  |  |  |  |  |
| All-cause | 17765 (195) | 13492 (147) | 14254 (154) | 13506 (147) | 12369 (133) | 11774 (124) | 11309 (118) | 11200 (115) | 13031 (130) | 12689 (125) | 11966 (117) | 14853 (143) | 15159 (145) | 15314 (145) |
| Traffic accident | 3135 (34) | 2874 (31) | 2409 (26) | 1340 (15) | 1898 (20) | 1267 (13) | 1307 (14) | 1288 (13) | 1556 (16) | 997 (10) | 1012 (10) | 1395 (13) | 1069 (10) | 1059 (10) |
| Falls | 5338 (59) | 3388 (37) | 3472 (37) | 4433 (48) | 3414 (37) | 4148 (44) | 3485 (36) | 4082 (42) | 4585 (46) | 4153 (41) | 4638 (45) | 6403 (62) | 5838 (56) | 5735 (54) |
| Struck by/against | 5476 (60) | 3659 (40) | 4018 (43) | 3823 (42) | 4012 (43) | 3703 (39) | 3946 (41) | 2648 (27) | 3298 (33) | 3850 (38) | 3076 (30) | 3965 (38) | 2902 (28) | 3398 (32) |
| Stabbing | 1545 (17) | 1077 (12) | 1494 (16) | 998 (11) | 897 (10) | 740 (8) | 258 (3) | 668 (7) | 538 (5) | 808 (8) | 602 (6) | 946 (9) | 1137 (11) | 830 (8) |
| Extreme temperature | 647 (7) | 1095 (12) | 1783 (19) | 1173 (13) | 1077 (12) | 575 (6) | 1033 (11) | 1240 (13) | 1597 (16) | 1808 (18) | 1028 (10) | 717 (7) | 2194 (21) | 2434 (23) |
| Poisoning | 584 (6) | 425 (5) | 259 (3) | 715 (8) | 395 (4) | 182 (2) | 361 (4) | 226 (2) | 206 (2) | 108 (1) | 436 (4) | 54 (1) | 565 (5) | 365 (3) |
| Others | 1039 (11) | 974 (11) | 819 (9) | 1025 (11) | 676 (7) | 1160 (12) | 920 (10) | 1048 (11) | 1251 (13) | 966 (10) | 1174 (11) | 1373 (13) | 1453 (14) | 1493 (14) |

* Values are presented as estimated number (hospitalization rate per 100,000 worker).
